# A New Concept to Secure Food Safety Standards against *Fusarium Species* and *Aspergillus Flavus* and Their Toxins in Maize

**DOI:** 10.3390/toxins10090372

**Published:** 2018-09-13

**Authors:** Balazs Szabo, Beata Toth, Eva Toth Toldine, Monika Varga, Nandor Kovacs, Janos Varga, Sandor Kocsube, Andrea Palagyi, Ferenc Bagi, Dragana Budakov, Vera Stojšin, Sanja Lazić, Marija Bodroža-Solarov, Radmilo Čolović, Goran Bekavac, Božana Purar, Djordje Jocković, Akos Mesterházy

**Affiliations:** 1 Department of Field Crops Research, NARIC, 6726 Szeged, Hungary; szabob@gabonakutato.hu (B.S.); beata.toth@gabonakutato.hu (B.T.); varga.j.monika@gmail.com (M.V.); 2 Cereal Research Nonprofit Ltd., 6726 Szeged, Hungary; zsigi2011@gmail.com (E.T.T.); nandor.kovacs@gabonakutato.hu (N.K.); 3 Department of Microbiology, Faculty of Science and Informatics, University of Szeged, 6726 Szeged, Hungary; shigsanyi@gmail.com (S.K.); andrea.palagyi@gabonakutato.hu (A.P.); 4 Faculty of Agriculture, University of Novi Sad, Novi Sad 21000, Serbia; bagifer@polj.uns.ac.rs (F.B.); dbudakov@polj.uns.ac.rs (D.B.); stojsinv@polj.uns.ac.rs (V.S.); sanjal@polj.uns.ac.rs (S.L.); 5 Institute of Food Technology, Novi Sad 21000, Serbia; marija.bodroza@fins.uns.ac.rs (M.B.-S.); radmilo.colovic@fins.uns.ac.rs (R.Č.); 6 Institute of Field and Vegetable Crops, Novi Sad 21000, Serbia; goran.bekavac@nsseme.com (G.B.); bozana.purar@ifvcns.ns.ac.rs (B.P.); djordje.jockovic@ifvcns.ns.ac.rs (D.J.)

**Keywords:** *Fusarium culmorum*, *Fusarium graminearum*, *Fusarium verticillioides*, *Aspergillus flavus*, resistance, mycotoxins, complex resistance to pathogens and toxins, food safety

## Abstract

Commercial maize hybrids are exposed to different degrees of ear infection by toxigenic fungal species and toxin contamination. Their resistance to different fungi and toxin relationships are largely unknown. Without this knowledge, screening and breeding are not possible for these pathogens. Seven- to tenfold differences were found in resistance to *Fusarium* spp., and there was a five-fold difference in ear coverage (%) in response to *A. flavus*. Three hybrids of the twenty entries had lower infection severity compared with the general means for toxigenic species. Three were highly susceptible to each, and 14 hybrids reacted differently to the different fungi. Differences were also observed in the toxin content. Again, three hybrids had lower toxin content in response to all toxigenic species, one had higher values for all, and 16 had variable resistance levels. Correlations between infection severity and deoxynivalenol (DON) content were 0.95 and 0.82 (*p* = 0.001) for *F. graminearum* and *F. culmorum,* respectively. For fumonisin and *F. verticillioides* ear rot, the Pearson correlation coefficient (*r*) was 0.45 (*p* = 0.05). Two independent isolates with different aggressiveness were used, and their mean X values better described the resistance levels. This increased the reliability of the data. With the introduction of this methodological concept (testing the resistance levels separately for different fungi and with two isolates independently), highly significant resistance differences were found. The resistance to different fungal species correlated only in certain cases; thus, each should be tested separately. This is very useful in registration tests and post-registration screening and breeding. This would allow a rapid increase in food and feed safety.

## 1. Introduction

Maize is one of the most important cereals in the world. This crop is a regular host of toxigenic fungi infecting the ears, which can cause very high losses in crop yield. In contrary to wheat where *F. graminearum* is the leading toxigenic species nearly everywhere, the situation is more complex in maize, where at least two leading species exist from *Fusarium* and *Aspergillus,* with similar significance, but different amounts in different years. 

**Pathogens:** Logrieco et al. [1] mentioned 19 *Fusarium* spp. of maize in Europe. However, the most important species in most regions are *F. graminearum*, *F. verticillioides*. Therefore, their control is needed in every corn production area. In Hungary, *F. graminearum* and *F. verticillioides* are the most important [2]. In drier years the latter species is dominant. In wet years, such as 1974, the number of species increased to 16, including *F. graminearum* (30%), *F. verticillioides* (27%), *F. culmorum* (4%), *F. fusarioides* (3%), *F. avenaceum* (1%), *F. sporotrichioides* (6%), *F. poae* (2%), *F. semitectum* (4%) and several others with 554 isolates. The dry year of 1975 saw a 28% occurrence of *F. graminearum*, 69% of *F. verticillioides*, and the rest made up 3% of the species represented by several entries of *n* = 645 isolates. The case is similar in all countries where data from *Fusarium* surveys exist (Mesterházy [3], Table 1). Because *F. moniliforme* was reclassified to *F. verticillioides* [4], the position of *F. graminearum*, *Gibberella zeae*) remained. Many new *Fusarium* spp. were described or reclassified, but these changes do not interfere with our two main causing agents. As more than 90% of the *F. graminearum* isolates of wheat belong to *F. graminearum stricto senso* [5], we focused in this study on this specimen. *F. boothi*, for example, produces nivalenol (2 isolates of the 29) [5]. In 2007, *A. flavus* occurred at a higher rate in Hungary, and aflatoxin-contaminated grain was also detected. In 2012, the aflatoxin contamination rate was high, and 2017 a smaller epidemic occurred. Due to global warming, the occurrence of aflatoxin in the field was predicted to reach significant rates in epidemic years; therefore, resistance to *A. flavus* was also chosen for testing.

**Environmental/weather conditions, toxigenic species and toxins.** The three main pathogens mentioned need humid and moderately warm weather to infect silks and later the cobs. Thereafter, *F. graminearum* needs humidity and warmth, but not very warm or hot weather, *F. verticillioides* needs warmer and drier conditions, and *Aspergillus flavus* needs the hottest conditions, especially for toxin accumulation [3]. This means that in some years, only fumonisin, DON or aflatoxin occur; in another years, all combinations are possible at a high risk level. Experience throughout many decades shows that high toxin contamination is associated with outbreaks of significant epidemics. From this we may conclude that susceptibility relies somehow on disease epidemics and toxin contamination as summarized by Clements and White [6], Mesterházy et al. [3], Munkvold [7], Reid et al. [8]. For this reason, breeding for resistance has become a significant goal to decrease both disease severity and toxin contamination. Several authors such as Boling et al. [9], King et al. [10], Chaing et al. [11], Cullen et al. [12] mention resistance to disease (no toxins were measured). Others concentrate on the resistance to toxin, such as Brown et al. [13] Bolduan et al. [14], others look at genetic factors being specific in toxin contamination [15,16] but do not consider symptoms, and others investigate both disease severity and toxin contamination together [17,18,19]. Menkir et al. [20] reported on germplasms with resistance to aflatoxin contamination. Looking more closely at the data, we see that the resistance to toxin and disease do not overlap in every case. We do not know whether this is due to some additional metabolic pathways beside resistance. Complete agreement does not seem to be apparent between resistance and toxin accumulation. Since toxins have in most cases undergone strict regulation in human consumption, and restrictions in animal husbandry have also been suggested; the task now at hand is to achieve a low toxin contamination level. It is not clear what the real relationships are, but many independent data confirm a rather good and useful correlation between resistance to infection severity and toxin contamination; see Mesterhazy et al. [3].

**Economic significance.** The harvested yield loss is regularly lower than the much larger problems caused by the toxins because farmers may suffer total financial loss when the yield contains toxins above certain regulated limits. Then, the grain can become unsuitable for food and feed. About one third (own estimation AM) of the total crop value was lost in 2014 in Hungary (about 330 million Euros), partly because of lowered prices because of higher toxin contamination, and partly due to losses in animal husbandry and additional costs of toxin binders, medication, etc. This epidemic again underlined the necessity of higher resistance levels in maize production. Nobody has exact data about losses in yield, value of the crop, or health costs in the human population and animal husbandry. However, we would be surprised if it would be globally lower than several billion dollars. Hungary and Serbia have had *Fusarium* problems for a long time [21,22] and selection programs have started with moderate success. This, combined with the appearance of aflatoxin-producing species, especially *Aspergillus flavus*, resulted in aflatoxin contamination above the EU limit in 2007, 2012 and at a lower degree in 2017. This alerted plant breeders and the milk industry in Northern Italy, Serbia, Slovenia, Croatia, Romania and in Hungary [23,24,25,26]. It seems that aflatoxin will not be a transitional problem we mentioned earlier. Battilani et al. [27] indicated a very strong predicted increase in aflatoxin contamination for nearly all of France, the whole of the Carpathian Basin and the Balkan area when the average temperature increases by 2 °C. At an increase of 5 °C, nearly all of mainland Europe except Scandinavia will become moderately or heavily contaminated areas. For this reason, this *Aspergillus* and aflatoxin problem should be taken seriously.

**Breeding aspects of resistance.** The most important fact is that at present, knowledge of complete resistance does not exist against any of the toxigenic species; what we have is a partial one at different degrees or none at all. In the past decades, most breeders favored natural infection. They were and are mostly convinced that this is the right way. They think that during the long years of variety breeding and testing, the probability is high enough to select the most resistant plants and hybrids. The variety registration and post registration were proved in the highly epidemic years; however, the disease pressure under natural infection is not enough to find the best ones—maybe it can be suitable to discard the most susceptible ones. We should add that the toxigenic species rapidly change from year to year. After a ‘*F. graminearum*’ year, we might have a ‘*F. verticillioides*’ or an ‘*A. flavus*’ year or any combination of them. As we do not have any proof that the resistance to different toxigenic fungi would be the same, the data speak against it, and natural infection does not allow a solid base for the breeding of more resistant hybrids. Therefore, most of the hybrids belong to the susceptible or very susceptible category. When we see the literature, in most cases, the breeding started only against the most important species. In best cases, two species are involved, but never more. It seems that the breeding and registration system could not adapt to the genetic background of the resistance.

**Figure 1:**
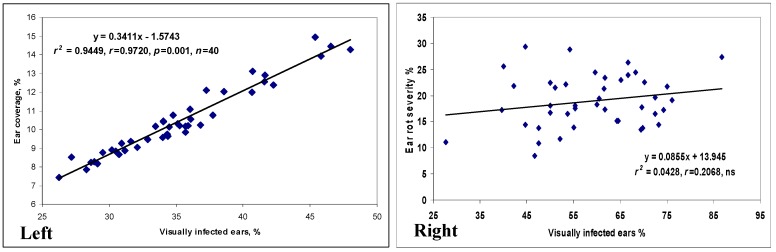
Natural infection of top maize hybrids in the post registration test organized by the National Alliance of cereal growers, performed by the National Variety Office (NÉBIH) 2010. (**Left** panel) Mean for eight locations, (**Right** panel) the most severely infected location, Eszterágpuszta.

In the cases of variety registration, only natural infection was considered in Hungary and elsewhere [3]. Severe epidemics were rare, but 2010 showed a nation-wide epidemic. The maize post registration test, sown this year and performed in eight locations (Figure 1) by the National Variety Office NEBIH, Hungary [28] with the support of the Association of Cereal Growers, Hungary brought important results. The mean data for infected ears from the total are given as a percentage, and the ear coverage is also rated as a percentage. The correlation is close, indicating that a higher ratio of infected ears correlates well with the infected ear surface. The most resistant hybrid had a 26.3% visual rate and 7.5% coverage (Figure 1, left panel). The toxin content was not measured, but such infection severity like this normally causes toxin levels to rise far above the toxin limit. When considering the most severe epidemic in Eszterágpuszta, the least infected hybrid had a 26% rate out of the total, and an 11% ear area showed visible infection. The maximum was 85% and 29.4%, respectively (Figure 1, right panel). The conclusion is clear: very resistant hybrids do not exist in this group. Therefore, the breeding efforts of various breeding firms are by far not enough to give farmers a chance to produce healthy grain when they face an epidemic that might not occur every year. The situation is slowly changing, however, because farmers look for better hybrids in this respect. The reason is simple: they are pressured to keep toxin contamination levels under control. The other important conclusion is that in spite of the lack of the hybrids in the most resistant regions, we have a significant variability in resistance indicating a utilization of these differences. With the withdrawal of the susceptible hybrids from commercial production food safety, there would be a sharp increase in food and feed safety. Then, the breeding efforts could result in further significant improvement in food and feed safety.

For the reasons above, artificial inoculation methods should be introduced and applied at a much higher level than previously used in registration and breeding.

The artificial inoculations are normally conducted with a pure isolate or a mixture of isolates, but a mixture of different species can also be the case [3]. In wheat, we have learned that the different isolates have different aggressiveness; therefore, gathering data during epidemics at different severity may help to produce more reliable resistance data [29,30,31]. For this reason, we wanted to test our hypothesis (more isolates of the same species are used separately to have more reliable results) in maize. Statistically significant resistance differences to *Fusarium* spp. [3] and *A. flavus* already exist [13,32,33,34,35], and all fungal species should be treated together (*Fusarium* spp., *A. flavus*) as they will remain important players also in the future.

A real answer to the question of the resistance level and resistance specificity or non-specificity cannot be expected from steadily changing natural infection and steadily changing fungal populations. Even though we have data about resistance correlations of toxigenic species in maize, from the toxigenic species, a maximum of two were involved, but not more. In several cases, a correlation was found between *F. graminearum* and *F. verticillioides*, and in other cases between *A. flavus* and *F. verticillioides* (see details in the review by Mesterhazy et al. [3], but not in every case.

What this means for us as researchers is that we should provide reliable data for the farmers and breeders on resistance behavior of the hybrids to all fungi. The data we have now show that relationships between resistances to different toxigenic species might be present, but not necessarily so. Therefore, we should test the hybrids separately against the most important toxigenic fungi to observe their resistance, toxin contamination and estimate their safety risks to individual pathogens and consider them together.

In this paper, our objectives were as follows: (1) To test the resistance of Hungarian and Serbian maize hybrids against toxigenic fungi such as *F. graminearum*, *F. culmorum*, *F. verticillioides* and *A. flavus*. (2) To test toxin contamination following artificial inoculation. (3) To test the use of more isolates separately. (4) To suggest a screening methodology that allows the preference of multi toxigenic fungal resistance in maize hybrids and thereby significantly improve food and feed safety.

## 2. Results

### 2.1. Ear Rot

**Figure 2:**
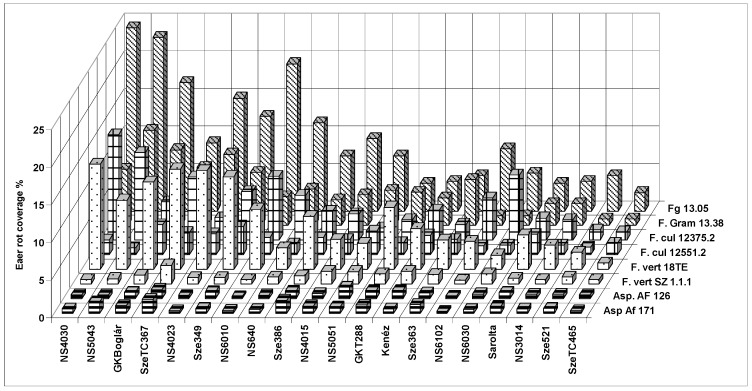
Expression of resistance based on ear rot data (coverage as %) of maize hybrids against toxigenic species and their isolates in maize hybrids against isolates of toxigenic species across years and locations, 2012–2013.

**Figure 3:**
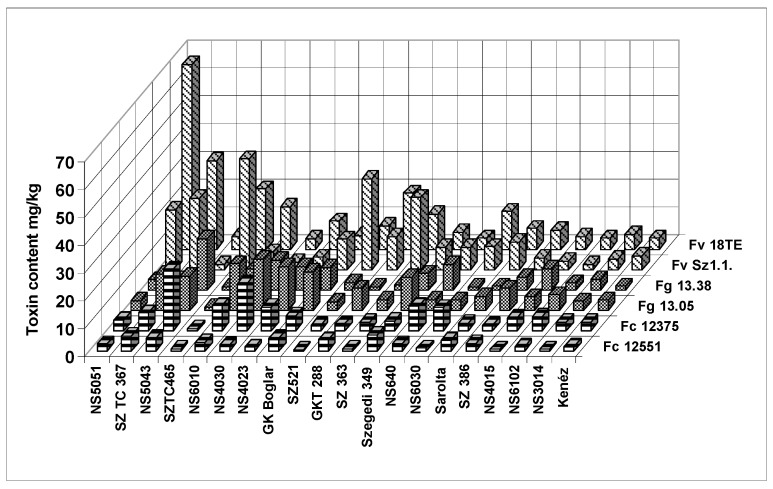
Toxin production of the tested toxigenic fungi and their isolates with respect to the toxin contamination of the hybrids with differing resistance to the toxigenic species across years and locations, 2012–2013. Toxins measured: ^x^ Fg = *F. graminearum:* DON, Fc = *F. culmorum:* DON, Fv = *F. verticillioides*: fumonisin B1 + B2.

**Figure 4:**
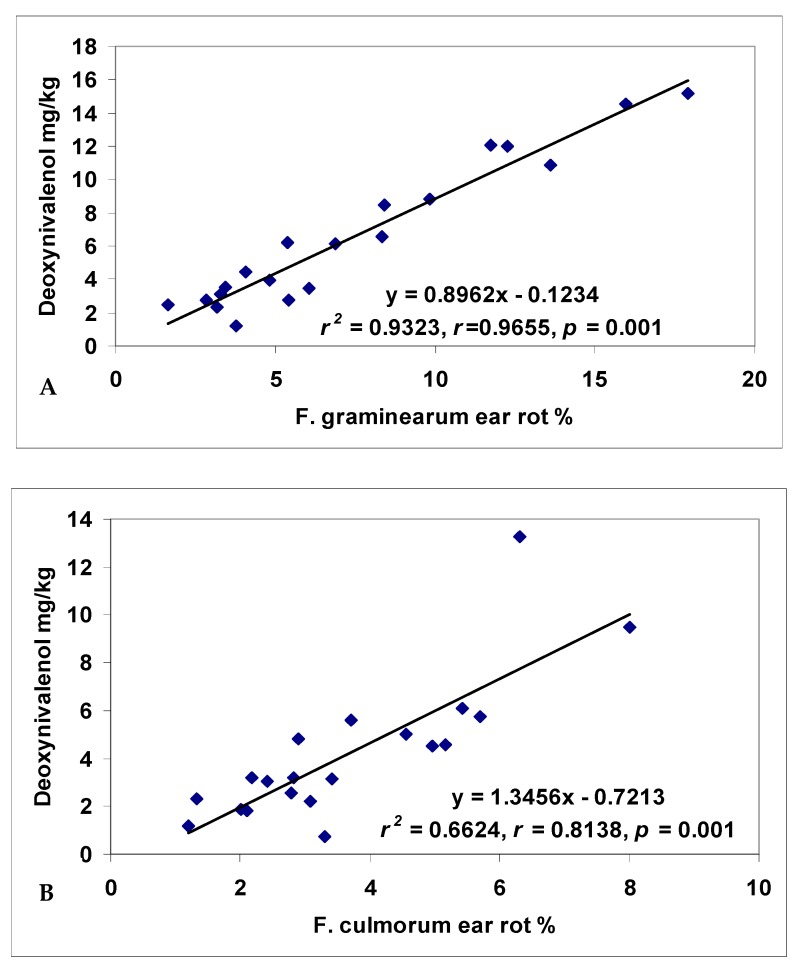

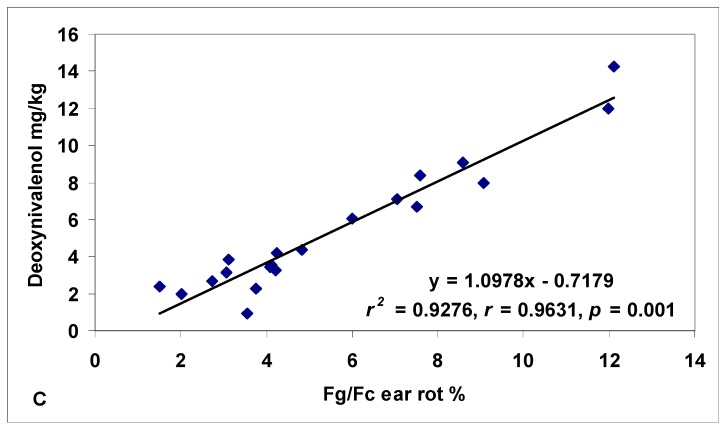
Regression relationships and correlations between ear rot and DON data for *F. graminearum* (**A**), *F. culmorum* (**B**), and both together (**C**), 2012–2013.

**Figure 5:**
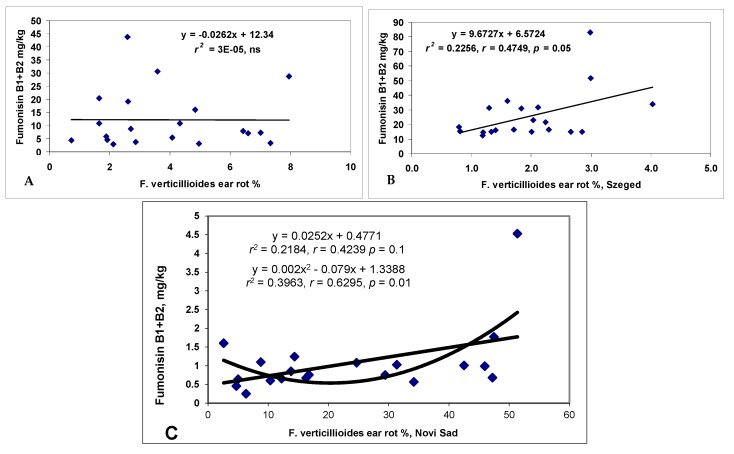
Regression relationships and correlations across years and isolates between pooled ear rot and DON data for *F. verticillioides* (**A**), Szeged (**B**) and Novi Sad /NS/ (**C**), ns = non significant, 2012–2013.

In this section, we aimed to compare the general means of infection to the different toxigenic species. Then, we present the ANOVA for the whole tests and also the ANOVA for every toxigenic species separately, and thereafter also the isolate specific data. As the dominant species dominate less pathogenic *F. verticillioides* and *A. flavus*, a Pearson range correlation test was also conducted to compare ranges and assess the stability of the genotypes to different toxigenic species. All tables and figures show results of two years and locations. This is true for the Figure 2, Figure 3, Figure 4 and Figure 5.

The artificial inoculation data (Table 1) show the mean ear coverage data for hybrid toxigenic species. The mean data show seven-fold differences. In this test the most pathogenic species was *F. graminearum* with an ear coverage of 7.4%. For *F. culmorum* and *F. verticillioides* and the least pathogenic species *A. flavus*, their mean values were significantly lower. This corresponds to the data in the literature with the difference found between *F. graminearum* and *F. culmorum*. The correlations between the resistances to different *Fusarium* spp. were significant. The resistance of *A. flavus* did not show any significant correlation with that of *Fusarium* spp., so a common genetic base can be excluded. The data show that because of the large differences between toxic species the general mean should be determined by using data of *F. graminearum*, which were ten times higher than the *Aspergillus* data. More importantly, the individual species showed closer relationships between their data and mean performance, but with no significance between the data from *Aspergillus* and the control data. For this reason, we measured the LSD to be 5% not only for the general means, but separately also for all toxigenic species and the control, as listed below in the mean data of Table 1. The lowest coverage values were found in three Szeged and one NS hybrids and in the most sensitive group, two Szeged and two NS hybrids were found. This means that in both variety groups we had a large variability among hybrids from the best to the worst. The highlighted data show the genotypes, which have a lower than the average value for the given trait. We have three hybrids where all data are below the mean and also three hybrids where all data show consequently higher infection than the mean. For the remaining 14 hybrids all possible variations occur. Counting the risk of the given hybrid, a check infection is considered like Sarolta that has a good position with respect to all toxigenic fungi, but the natural infection is higher than the mean. The best case is when all toxigenic fungi show a low value and also the control values correspond to them, e.g., for SzeTC465. NS4030 is highly susceptible to all three *Fusarium* spp., but has extremely high resistance to *Aspergillus* and natural infection.

The ANOVA (Table 2) shows the influence of the main effects and their interrelationships. The main effects and their interactions are all significant between *p* = 0.001 and 0.05. As interactions from five-way ANOVA for 3, 4 and 5 interactions are hard to interpret, we pooled (both SS and df-s) them into one common interaction that can be seen in Table 2. The main effects were all highly significant, but we can estimate their relationships better from their interactions. For us, the interactions with hybrids are especially important (Table 2). We see that all interactions with hybrids are much lower (more diminished) than the hybrid main effect. The hybrid main effect is superior to the A × B interaction at a significance level of *p* = 0.001. We showed a significant difference between Hybrid effect (A) upon the Hybrid × Isolate (A × B) interaction, and Hybrid effect A upon Hybrid × Toxic spp. (A × E) interaction. This means that even though the pathogenicity has a modifying effect, the decisive factor is the resistance level. For the year and location, the MS values do not differ significantly from the corresponding interactions; therefore, dominance of the main effects over these interactions cannot be determined.

The ANOVA test, however, was only partially informative for the individual toxigenic fungi as the difference in aggressiveness was very high between them; the LSD was determined by the high *F. graminearum* values. For this reason, we also performed ANOVA for them separately. The results show (Table 3) a similar pattern we had for the general ANOVA, but there were also differences. The main effects were significant for all *Fusarium* spp., but in *A. flavus* the location and isolate did not differ significantly, e.g., their response was not different. Thus, stability seems to be higher, and the values were less influenced by isolate and location. For *F. graminearum* the hybrid effect was 1.5–4-fold higher than the two-way interactions with the hybrid component, which seemed to be more stable. In *F. culmorum* the interactions were larger than the hybrid effect, so the hybrid reaction is less exact. *F. verticillioides* is closer to *F. graminearum* as interactions (AB–AD) are lower than the hybrid effect but this does not reach a difference that could be considered to be significant. The location, year and isolate effects compared to the A × B, A × C and A × D interactions show a significant difference compared to the interactions in all *Fusarium* species, but not in *A. flavus.* When considering the interactions at different toxigenic species, it is clear that the responses were different, e.g., we do not see a pattern that would be valid for all fungi. The Within values show large differences (1.12–36.7); therefore, a common analysis is possible as shown, but the species-specific ANOVAs could be performed to give a very clear picture of the data and cannot replace a general analysis as shown in Table 2.

The isolate specific data (Figure 2) clearly show that the isolates of the same species do not automatically reflect the same or similar data. This is also reflected in the correlation values (see Table 4). This means that we cannot obtain highly repeatable genetic results for a single inoculum. In *F. graminearum* there is a good agreement, despite the existing differences. For *F. culmorum* and *F. verticillioides* no significant correlation was found, e.g., the reactions to the two isolates strongly diverged. In *A. flavus* a medium level of correlation was observed. In 2012 *A. flavus* was recorded at an epidemic amount. Thus, performing the *Aspergillus* resistance test became quite relevant and was introduced based on the observations from 2007. It is interesting that *A. flavus* AF.126 reacted very similarly to *F. verticillioides* Sz 1.1.1 (*r* = 0.729, *p* = 0.001). *F. verticillioides* Sz.1.1.1 had a lower, but significant correlation of *r* = 0.546 (*p* = 0.01) with the other isolate of *A. flavus* (171). However, the data of the *F. verticillioides* isolate 18TE did not have any significant relationship with *A. flavus* isolates. The data mean that we have no solid basis to accept the results of a single isolate as genetically valid resistance data against any toxigenic species. In *F. graminearum* the tendency is similar, but the two isolates of *F. culmorum* and *F. verticillioides* do not show any significant correlation for resistance. *A. flavus* isolates reached a medium level significant relationship (*r* = 0.530, *p* = 0.02), so a general pattern does not exist.

Spearman’s range correlations. Regarding the correlation values of the original toxic species data (Table 1), we noted that the correlations between resistances to different fungal species were very different (Table 5). In order to get a clearer picture about the individual positions of the given hybrids, we calculated the ranks of the 20 genotypes and their correlations. The mean ranks across fungal species varied between 2.2 and 18, indicating a cc. nine-fold difference in mean performance. Two hybrids, specifically SzeTC465 and NS3014, produced lower than 10 ranks compared to all toxigenic species and the control as well as the general mean values across fungal species. On the other hand, three hybrids had above average susceptibility values compared to all toxigenic species. In these cases, the variance was also low (1.7–6). Each of the 15 remaining hybrids showed mixed performance with data, which were both lower and higher than the mean value. For example, NS6202 was superior with respect to Fg but not Fc. Sarolta had a good general performance except against *A. flavus*. NS 640 showed susceptibility to *F. graminearum* and *F. culmorum*, but the resistance to other species was good. In NS4030, we had high susceptibility to *F. graminearum*, *F. culmorum*, and F. *verticillioides*, but excellent performance against *A. flavus* and natural infection (check). This means that we should prefer those hybrids that perform well with every toxigenic species and also under natural infection. It also means that all traits should be considered for making a good decision based on the values of the given hybrid. The correlations show a similar picture compared to what we had for the original ear coverage data, but the main difference is that the low value of mean data from *A. flavus* and natural infection (check, UTC) also gave a significant correlation with the mean performance.

The hybrids not bred for resistance against toxigenic fungi revealed 6–10 fold differences in resistance. This means that we have a wide variability to each pathogenic species to utilize it for the farmers. The resistance does not seem to be related to the most important pathogens, but we have three hybrids that show lower than average resistance to each pathogenic species, including natural infection. The Spearman range correlation was suitable to analyze the different or similar response of a given hybrid to different pathogens. The hypothesis that isolates will bring different or maximum similar results to different pathogens could be supported also in maize. 

### 2.2. Toxin Data

In this chapter we had the same goals as for the 2.1 task.

The toxin data across isolates, years and locations (Table 6) show highly significant differences in the hybrids compared to all toxigenic species. Unfortunately, the two *A. flavus* isolates did not provide any useful data for aflatoxin contamination in Szeged and Novi Sad (even they were controlled for aflatoxin production in vitro with positive result); therefore, this aspect could not be analyzed. The maximum and minimum values differed 7–16 fold for the different toxins, where the DON of *F. graminearum* value was lowest. We made produced the toxin contamination from the non-inoculated control, but all data were 1–2 below the detection limit. Therefore, they are not shown. There are seven genotypes which showed a lower performance against all toxigenic species compared to the average (highlighted with yellow). This is the double number we found for ear coverage percentages. We have only one genotype where all data are worse than the average. Altogether, this covers eight of the 20 hybrids having similarly good or poor resistance to the pathogens considered. This is similar to what we observed for the ear rot data, although individual hybrid reactions might differ in toxin production. For example, the highest toxin contamination was found for *F. verticillioides*, where the mean toxin contamination was twice as high as the DON for *F. graminearum*, but in ear rot, the *F. graminearum* data were twice of the value of the *F. verticillioides* data. The close correlation between *F. graminearum*/*F. culmorum* for the ear symptoms was also supported here. However, correlations between *F. verticillioides* and *F. graminearum* as well as *F. verticillioides* and *F. culmorum* were not significant and in general, were negatively correlated with each other. Thus, we should call into question the close phenotypic correlation between the ear rot indices to have a selection value. In the visual symptoms, *F. graminearum* played the same role.

According to the ANOVA calculated separately for each toxigenic species (Table 7), the hybrid effect was significant for all three *Fusarium* spp., and the Szeged and Kupusina data again differed significantly. The ‘year’ effect was significant, except for *F. culmorum*, and the differences between isolates were also significant, except for *F. verticillioides*. The two-way interactions containing the hybrid effect were smaller than the genotype effect, but the difference between them was seldom shown to be significant. The visual symptoms showed a higher difference and higher stability. There is also proof that ear rot and toxin responses are not equivalent.

Here, the ranks were also calculated (Table 8). Only five genotypes were found which showed a similar performance to different toxigenic species; three of them belonged to the most resistant group, having very good general resistance. Again, three were found with similar stability on the susceptible side. In the other 14 hybrids, different reactions were characteristic. Within the Szeged and Kupusina at Novi Sad genotypes, extremely good and bad performances were both found. Among the hybrids highly susceptible to *F. graminearum* and *F. culmorum* we found extremely low fumonisin concentration, such as in NS6010 and NS50143, or very low concentration in the response of NS4030 to *F. verticillioides*, but very high for *F. graminearum* and *F. culmorum.* However, we also have good examples for *F. graminearum* and poor ones for *F. verticillioides* toxin production (Sze521 and Kenéz). The correlations were surprising, in that the fumonisin data showed non-significant negative correlations with the DON data while the ear rot data were significantly and positively correlated.

We also checked the differences between the responses of the isolates of the same toxigenic species (Figure 3). The figure clearly shows that the two isolates of the same fungal species often show different toxin contamination in a given hybrid, similar to what we had observed in the ear rot data. The highest toxin contamination was recorded in fumonisin at relatively low ear rot values. The correlations between reactions to different isolates showed a variable picture (Table 9). For *F. verticillioides* and *F. graminearum* isolates we found moderate, but still significant correlations, but for the other *F. culmorum*, a significant correlation was not found. These data will have consequences for the methodology of testing resistance. The correlations between the inocula of the same species are also divergent, supporting the view we formed based on the visual ear coverage data. 

The data prove that the toxin responses differ from the symptom severity (ear coverage %), in spite of the similarity.

### 2.3. Relationships between Ear Rot and Toxin Contamination

It is important to note how much resistance can contribute to the reduction in toxin levels. For this reason, the general means were compared between ear rot and toxin contamination from the artificially inoculated treatments. As *F. graminearum* and *F. culmorum* are closely related and their toxin spectrum is also very similar (DON, zearalenon), we treated them together. *F. graminearum* was in this test more aggressive and reached higher DON contamination than *F. culmorum*. Therefore, we were also interested in the relationship between the two species. The *F. graminearum* ear rot /DON correlation was *r* = 0.9655 (Figure 4). The correlation between *F. culmorum* ear rot and DON is somewhat weaker, but the correlation value of *r* = 0.8138 is highly significant (*p* = 0.001). The source of the difference is most likely due the lower infection level that allowed less differentiation of the hybrids. The pooled ear rot and toxin data for the two species were correlated at a Pearson correlation of *r* = 0.9631, which corresponds to a robust correlation. All are significant at *p* = 0.001.

*F. verticillioides* showed a different picture (Figure 5). The general means did not show any significant correlation between ear rot and the fumonisin content (*r* = 0.0162, ns). The two locations produced very divergent data, with Szeged producing lower ear rot data, but rather high fumonisin contamination, Kupusina gave higher ear rot data values, but the fumonisin contamination was much lower. For this reason, we performed the analysis separately for both locations. Both correlations were significant, both with *r* = 0.47, *p* = 0.05. Of course, this will severely influence resistance data and their exactness. The results also show that the ecological conditions favoring disease and toxin accumulation might be very different. 

The *A. flavus* infection was rather close in the two locations, but in Szeged and Kupusina no aflatoxin contamination could be measured. 

It is important that visual rating and toxin contamination do not always agree; we can see that best on the comparison of the ranges of symptoms and toxin contents. It seems that toxin contamination does not reflect always visual rates. Therefore, without toxin data, a correct evaluation of the plant material is not possible.

## 3. Discussion

### 3.1. Resistance Relationships

The resistance problems of maize to ear rot pathogens are rather complex. We have seen that the natural infection cannot be screened effectively enough, as shown clearly by the data from the variety office. Therefore, artificial inoculation remains as the only alternative. The recent literature has shown the need to test maize genotypes for resistance not only for one dominant causative agent, but also for two (see details Mesterhazy et al. [3,14]). Correlations, however, vary. However, in Hungary we face four such causative agents; therefore, in order to decrease the toxin risk, we need to treat all of them. As resistance to *F. graminearum* and *F. culmorum* is very similar, and our earlier results also came to the same conclusion with regards to other set of hybrids [36,37,38,39], we have the chance to choose between them. As the significance of *F. graminearum* increases greatly in the north, similar to Germany and Northern Europe [39,40], it seems that the resistance testing to *F. graminearum* only would be adequate. This problem, however, should be decided in situ as there are large ecological differences within the northern European regions. As *F. culmorum* prefers cooler temperatures, its use during cooler years might be advantageous. 

We see that the resistance to *F. verticillioides* correlates well at the symptom level with that of the *F. graminearum* and *F. culmorum* data. The *A. flavus* data do not show any significant correlation with any of the *Fusarium* spp. data. Additionally, we need a more reliable way of evaluating resistance. In wheat we have common resistance to different *Fusarium* spp. [22,41]; in maize, the situation is more complex. Presello et al. [42,43] found good correlations between *F. graminearum* and *F. verticillioides* resistance for the tested genotypes. Löffler et al. [44] found good phenotypic and genotypic correlations between *F. graminearum* and *F. verticillioides* resistance. There are data that *F. graminearum* and *F. culmorum* resistance are closely related [36,45] Henry et al. [19] compared *F. verticillioides* and *A. flavus* resistance. They found an *r* = 0.72 correlation (*p* = 0.02) between the reactions to these toxigenic species. Williams and Windham [46] (2009) found useful resistance relationships between *A. flavus* and *F. verticillioides*. The results clearly showed that highly significant differences can be presented against all toxigenic species. However, the resistance to the different agents showed a variable picture. There were three hybrids in this test that showed good resistance to all pathogens. We again had several hybrids that were susceptible to all pathogens, but the majority responded differently. The close *F. graminearum* and *F. culmorum* correlation was demonstrated again. The *A. flavus*/*F. verticillioides* correlation was not significant in our study, and this was valid for the correlations between *A. flavus* and *F. graminearum* as well as *F. culmorum*. Robertson-Hoyt et al. [47] showed a significant correlation between *F. verticillioides* and *A. flavus* and they presented evidence for this from QTL analysis [48]. Xiang et al. [49] performed a meta-analysis from 14 different studies for the three main ear rot pathogens (*F. graminearum*, *F. verticillioides* and *A. flavus*) and found QTLs on the same chromosomes indicating the possibility of a common resistance to these pathogens. In our material these findings could not be supported, but the two results do not exclude each other, as in hybrids with different background this can be true. When taking the resistance data into account, a general agreement does not exist between resistances to different pathogens. However, several cases were found where such a common resistance and lack of all resistance to these pathogens may exist. Most of the hybrids reacted differently to one or more pathogens, in our study about 80%. We could not draw general conclusions, as in different maize hybrid populations the results are also different (Mesterházy unpublished).

We should say that these resistance correlations are not generally valid for every tested hybrid set. We tested a large number of hybrids, and we often got variable resistance in different hybrid populations, so it cannot be stated that other findings, showing a closer correlation between *A. flavus* and *F. verticillioides* would not be possible, since we have identified hybrids with resistance to both. Another question is the genetic base of this phenomenon. As in Szeged and Novi Sad, no such genetic work was carried out, so in this respect no information exists. However, these and other tests may help later breeding efforts to increase resistance. We can say as the majority of the hybrids show different resistance levels to the main pathogens, we should adapt to this and test separately the resistance to these in our hybrids. The inclusion of ranks did not fundamentally change the correlation matrix, even though smaller modifications did occur. However, the variance of the ranking gave a more precise insight into the relationships between resistances; it serves some art of stability index. It was an important lesson that because of the large pathogenicity differences between pathogens, the LSD 5% values should have been counted against all pathogens differently and the mean infection severity has only secondary significance. On the other side, the large variability in resistance found can be utilized in various registration and post-registration studies to increase food and feed safety.

### 3.2. Toxin and Resistance

Infection severity and toxin contamination are major issues. The real question is how relevant the resistance level is for toxin contamination. Henry et al. [19] found significant correlations between *F. verticillioides* and fumonisin contamination and similar results were also found between *A. flavus* ear rot severity and aflatoxin level. If we can confirm a stable and very close correlation, we can then declare resistance to disease and resistance to toxin to be interchangeable synonyms. Bolduan [14] found a Pearson correlation of *r* = 0.94 between *F. graminearum* infection level and DON contamination. We can conclude that resistance is surely an important factor. 

The present study found a correlation value of *r* = 0.9655 (*p* = 0.001) for *F. graminearum* resistance and toxin accumulation. We had very similar data compared to Bolduan [14]. The correlation between resistance and toxin data for *F. culmorum* showed *r* = 0.81, and the pooled data of the two species showed again a correlation of *r* = 0.96 (*p* = 0.001). These correlations in this study are much higher than most of the data; however they correspond well to the data of Bolduan [14]. Of course, this does not mean that all hybrids fit into this scheme, since in recent years we found very divergent hybrids, but not in high numbers. It seems, however, that the close correlation for *F. graminearum* fits. For *F. verticillioides* the numbers are lower, and the pooled data from the two locations did not show any significant correlations. However, a significant correlation was calculated at both locations separately. It seems that a given amount of visual ear rot infection may result in a very different level of toxin contamination. The *F. verticillioides* test showed that the different locations produced contradictory data, and even negative correlations were found compared to the data of the other toxigenic species. In visual symptoms we found a significantly higher than average correlation between *F. graminearum* and *F. culmorum*, but this positive correlation became negative and non-significant when toxin contamination was compared with a single isolate. On the other hand, in Szeged, the relatively low infection data produced very high fumonisin contamination. In Novi Sad, the much higher infection numbers resulted in only a low fumonisin content which was not enough to differentiate strictly between the genotypes. This shows that ecological conditions for disease development and toxin production are not necessarily the same, as can be seen from the data that we presented.

**Figure 6:**
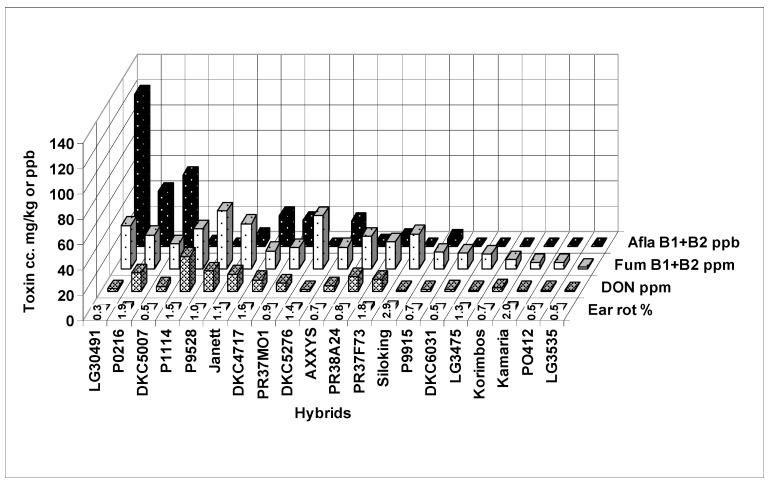
Natural toxin contamination of maize hybrids 2014, MKK test, Szeged, Hungary.

In *F. graminearum* we have a special symptom that cannot be seen on unshelled ears. We know from Christensen and Kaufmann [50] that the *Fusarium* stops spreading on the kernels at 23% grain humidity. However, at this stage the cob normally has 30% or higher water content or higher, so the spread of the fungus continues until the cob dries out [37]. This means that a spreading time of about two weeks should be added to the cob compared to grains. Visual symptoms 40 years ago were not developed; only the seed germination test showed very high *F. graminearum* infection. When the cobs were examined after shelling, an extended grayish zone was observed, sometimes covering 50–60% of the cob area. We first observed this in the seventies, and also recently in 2014. The September and October months (2014) were very rainy; the cobs were visually only marginally infected, but about 10% of the kernels showed the reddish to purple discoloration and mycelium of *F. graminearum*. Furthermore, most of the top of the kernels were healthy. Oldenburg and Ellner [39] also confirmed this finding. In the hybrids, natural toxin contamination was measured (2014). Figure 6 shows clearly a very important finding. The visual natural infection was low, 0.3–2.9%. We did not see diverging ear rot values DON between 0 and 27 mg/kg; fumonisin B1 + B2 was found between 1 and 46 mg/kg, and aflatoxin between 0 and 121 g/kg. Of course, a possible reason for the genotype deviation may be considered. We wished to demonstrate that a very low visual symptom severity can lead to very different levels of toxin contamination, sometimes also for fungi that could not be observed by simple visual evaluation. Since the summer was not hot, and September and October were rainy and cool, we did not reckon the strong presence of the aflatoxin and fumonisin toxins. It seems that we were wrong. This means that we should get a better understanding of the development of the disease and toxin.

**Figure 7:**
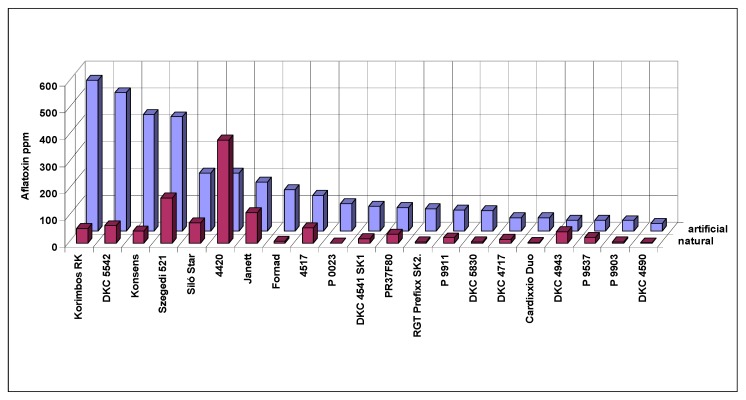
Aflatoxin contamination from natural and artificial resistance tests in maize, 2017, MKK test, Szeged, Hungary (artificial aflatoxin data are means of two isolates).

2017 was rather dry and hot; no significant DON (mean 0.24 mg/kg) and fumonisin (1.82 mg/kg) was found in the MKK resistance test for natural infection. However, aflatoxin contamination was very strong (Figure 7). The maximum value was 385 g/kg for natural and 562 g/kg for the artificial inoculation. The mean is 51 and 157 g/kg for the natural and artificial inoculation, respectively. Visual infection was 0.47% ear rot (0.02–0.82%). This means that the visual ear rot rates were nearly the same in the two years, but aflatoxin means increased from 15.69 to 58 g/kg. Three hybrids were identical: Janett increased from seven to 114 g/kg, DKC4714 started from 24.5 g/kg and decreased to 14 g/kg, and Korimbos was 0.10 g/kg in 2014 and reached 562 g/kg in 2017. This shows clearly we do not have a pattern that would say that an increase, for example, in temperature, drought, etc. increases or decreases aflatoxin by x%. As a mean this might be true, but for the individual hybrids it does not say anything. DKC4714 is very interesting. In spite of the much more favorable conditions it had a lower 2017 aflatoxin level than under the not favorable 2014. Of course, this a problem in phenotyping and might have serious problems in genetic work where we need a high accuracy. We should say that we have very limited or no information about the genetic and other causes of the changes in aflatoxin production, especially for individual hybrids. We have, however, several hypotheses to test to improve reliability of the data. The only possibility is now that the given hybrid must have low values in all tests to classify them to low, medium or high-risk category. Therefore, we need more data to exclude artifacts. This is valid for both artificial and natural data.

Battilani et al. [27] shows that North Europe is free, but Southern Germany and Czech Republic can have traces. For this reason we should take very seriously this message, and when we can breed and identify hybrids like DKC4714, we can avoid a lot of problems in the future. We should have hybrids with low risk to all important toxigenic species. As the data indicate, this is possible.

The hypothesis is that even resistance seems to be the strongest regulator of toxin production although other effects may also significantly influence toxin production. After all, our conclusion is that natural or visual rating of the hybrids is by far not enough to estimate their food or feed safety risks, so for this, the toxin contamination must be measured directly. As the market is directed by the toxin levels, we should adapt to this need.

### 3.3. How Much Inocula Should be Used?

From the literature, it is clear that everybody uses pure isolates in resistance tests, or a mixture of isolates of the given pathogen or a mixture of different species, but in all cases only one inoculum is applied [3]. At present we do have strong evidence in *F. graminearum* that no vertical races exist in wheat [22,41,51,52]; with a high probability this is valid also for maize as they are very closely bound in the epidemic cycles. We do not know about specific races in *A. flavus* and *F. verticillioides*. However, the similar testing results of many materials in different continents or regions with local isolates make it probable that this is rather a theoretical danger. For this reason the local isolates of these pathogens are enough to produce reliable results with not local use, too. Regardless, clear proof should be delivered to clarify this basic problem for breeding and testing. From this study it is clear that neither of the two used isolates of the four toxigenic species behave in the same way; in most cases significant differences exist in aggressiveness, and also the ranking of genotypes may be diverse in response to different isolates of the same pathogen. Therefore, any reaction to a given inoculum cannot be considered as genetically meaningful data, even though it is the mean of several replicates. The use of the mean reaction to four isolates was successful in wheat [30,31], and also gave better results (higher correlation values) than only one would have given as shown in the comparisons between Table 5 and Table 9 as well as in Table 2 and Table 7. Here, it should be mentioned that the isolate x year interaction is highly significant; therefore, a stable aggressiveness across years is not the case. For this reason, for cultivar registration or post registration studies we need at least two independent isolates, but it would be better to use three. This is therefore very important as only Szeged performs resistance tests use two independent inocula. The environmental interaction between the two isolates is non-existent as they are exposed to the same ecological conditions and so the result is closer to the genetic value. However, as we have observed in differences between years and locations, at least two years of study is necessary.

### 3.4. Testing and Breeding Aspects

Resistance is polygenic, determined by more or less QTLs [3]. This genetic background is stable; what differs is the resistance expression that can be variable under different conditions, years and isolates. The first task is to advise farmers in choosing the least risky hybrids for food and feed safety. We need to inoculate separately with the different toxigenic species and at least two inocula per species should be used, which can be either single isolates or mixtures. They should represent different epidemic severities, which helps to better predict the resistance level of a given hybrid. Based on the infection and toxin data, we can perform the risk evaluation of the hybrids for all fungal species, for example, low, medium and high risk. We did not find any hybrid with total resistance to any of the pathogens; therefore, at present, no hybrid in the no-risk category could be presented. However, the 5–10 fold differences between maximum and minimum severity and toxin contamination show a significant variation that can be exploited. Of course, the separate inoculation and use of more isolates is a basic task for the breeding to be able to deliver better hybrids to the market to increase food and feed safety in the long run.

## 4. Materials and Methods

### 4.1. Experimental Design and Plant Material

For the test, ten Hungarian hybrids from GK-Szeged and 10 hybrids from the Institute of Field and Vegetable Crops, Novi Sad were included. The Szeged experiment was installed in the Kiszombor experimental Station, in the Maros river valley. The Kiszombor station has alluvial soil with a high clay content. The humus content is 3%, with some variation in the field.

The previous crop was wheat. The sowing time was 30 April (2012), and 2 May (2013). 2012 was extremely dry; therefore, the nursery received irrigation (30 mm) just after sowing, thereby enabling a complete germination. Then, again at the middle of June, 40 mm water was given and after finishing the inoculations at the beginning of August (40 mm) to enable regular growth. In July and August there were three weeks when the temperature was above 35 °C. Our nursery survived well; plant habit and ear development were normal. 2013 was cooler, with only two irrigations before tasseling and another one at the end of July to have well-developed uniform stands. 2014 was rather dry: after sowing 30 mm rain was given, before tasseling another one, but September and October was very rainy with 200 mm rain. 2017 germination was complete: one irrigation was enough in middle June (30 mm), two smaller rainfall events (10–20 mm) in mid-July and beginning August helped the vegetation. The Serbian experiment was set up in Kupusina, close to the river Danube. Soil conditions were similar to those at Kiszombor, as well as environmental conditions except that temperatures in 2012 was 1–2 °C higher than in Szeged, whereas 2013 was also cooler with a less severe drought problem. This trial was irrigated twice each year, after sowing and before tasseling.

The experiment was carried out in a four replicated randomized complete block design. A plot consisted of 9 rows, 4 m long, a row spacing of 75 cm, and plant spacing of 20 cm. This meant about 20 plants could be planted per row. 4 × 2 rows served for inoculation with the two isolates of the four toxigenic species *F. graminearum* (13.38, 13.05)*, F. culmorum* (12,375, 12,551), *F. verticillioides* (18TE and Sz.1.1.1) and *A. flavus* (126 and 171). Each row was inoculated with a different isolate. All isolates of the toxigenic species were checked before increasing aggressiveness. In *F. graminearum*, *F. verticillioides* and *F. culmorum,* the toxin production mostly depends on aggressiveness, so direct toxin production control was not done. For *A. flavus,* both were necessary, as a significant part of *A. flavus* isolates cannot produce aflatoxin since the gene cluster for it is either missing or incomplete [4,6,24,26,33,53]. All isolates are single spore cultures and were identified by morphological and sequence-based methods. The last row was not inoculated as it served as a control exposed only to natural infection.

### 4.2. Inoculation and Evaluation of Disease

**Figure 8:**
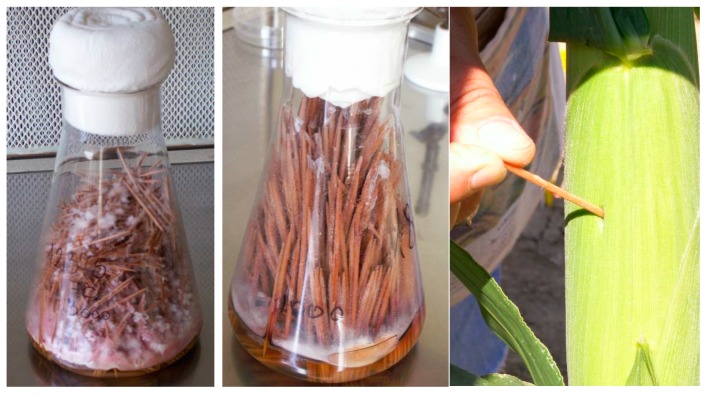
Toothpicks ready to inoculation, (**left**) *F. graminearum*, (**middle**) *F. verticillioides*, (**right**) Inoculated ear, inserting the toothpick in a hole 15 mm deep and 1.5 mm wide.

We preferred this method because mid-ear inoculation gave more stable results [44] than the silk channel method and also because our similar findings (unpublished) showed similar results. Furthermore, the severity of the disease is higher, and the differentiation of the hybrids is better. We used it for all toxigenic fungi. Otherwise the comparison of the resistance levels to different fungi can be problematic. For inoculation the toothpick method of Young [54] was used, modified by Mesterhazy [32]. The toothpicks were boiled with ion charged water each time for two hrs. from the start of boiling. When the color of the water after the third boiling had some color, a fourth boiling followed. This was important as tannins and other compounds in the wood inhibit fungal growth and they should be removed. Then, the toothpicks were air dried; depending on the number of plants to inoculate, smaller or larges flasks were filled with the necessary number of toothpicks with 10% plus as reserve. The flasks were then filled with liquid Czapek-Dox medium for 2 h. Then the medium was poured out except for 5 mm thick liquid to provide humidity for the fungal growth. The closed flasks were autoclaved at 1.20 bar pressure for 2 h. After cooling they were inoculated from test tubes grown from revitalized isolates from the freezer. In three weeks the inoculum was ready to use (Figure 8). We used single spore cultures and all inoculations were made separately. This means that the 8 rows of a 9 row plot were inoculated with 2-2 isolates of the toxigenic fungi with one left untreated for natural infection. The isolates were stored in lyophilized form at 80 °C. The same isolates were used in both years and locations to produce comparable results.

Inoculation was performed 6 days after midsilking for all fungal species. Only the upper ear was treated. A hole was made with an awl with a point (15 mm long and 1.5 mm wide) 6 days after midsilking, and then an infested toothpick was inserted and remained there until harvest. This was generally done 7–8 weeks after inoculation. Only those ears were harvested that had the toothpicks. Insect damaged ears were not considered during evaluation. We reported two data values, the first being the extent of infected area around the infection point, and another rate was the rate of infection we have seen on other parts of the ear. It happened that a secondary *A. flavus* infection developed on the *Fusarium* infected ear, this also was evaluated. In the un-inoculated control both *Fusarium* and *Aspergillus* were rated. Evaluation was given as the percentage of ear coverage. In an average hybrid ear we have about 750–800 grains. If we see only one diseased kernel, this corresponds to a coverage of 0.1%. When we see 7–8 infected grains that is 1 percent. *F. verticillioides* and *A. flavus* are weak pathogens; therefore, an infection level of 10% for the first is unusually high; in *Aspergillus* 1.5–2% (about 14–15 grains per ear) this is also unusually high, and seldom occurs. This way the infection severities are comparable for all pathogens. For toxin analyses, five average infected ears were chosen without insect damage. They were collected in a polyethylene webbed Rashel bag and left under dry conditions for three weeks. Then the five ears were shelled and the cc 1 kg grain was roughly ground and carefully mixed. From this 30–40 g was taken out and six g was fine milled by a Perten laboratory mill (Type: 3310, Perten Instruments, 126 53 Hagersten, Sweden). This way the sampling error could be reduced significantly.

### 4.3. Toxin Measurement

Five ears with average ear rot prevalence per row were selected for toxin analysis. The amount of DON was measured by HPLC, Agilent Infinity 1260 (Agilent Technologies, Santa Clara, CA, USA). The method has been described by Mesterhazy [30]. The fumonisin and aflatoxin contamination was measured by LC/MS/MS [55]. In Novi Sad, the determination of DON was carried out by HPLC-DAD, Agilent 1100 Series. Colum Zorbax SB-C18 (3.0 × 250 mm × 5 μm), method Matić et al. [56]. The samples for fumonisin B1 + B2 were analyzed in Szeged. Aflatoxin determination was carried out by HPLC UV light-FLD (Agilent 1100 Series, Agilent Technologies, Santa Clara, CA, USA and UVE LCTech GmbH, Santa Clara, CA, USA), Colum Zorbax SB-C18 (3.0 × 250 mm × 5 μm) followed the description of Ghali et al. [57] and Liu et al. [39,58].

### 4.4. Statistical Evaluations

To evaluate results, three, four and five-way analyses were performed with the help of the functions provided by Sváb [59] and Weber [60]. Beside this, correlation and regression analyses were performed using the built-in functions in Excel. The further evaluation of main effects and interactions is based on Weber [60].

## Figures and Tables

**Table 1:** Ear rot coverage as a percentage of maize hybrids in the resistance tests; 2012–2013 across years, locations and isolates.

Hybrid	Toxigenic Species, Ear Coverage %					Mean
	Fg ^x^	Fc	Fv	Afl	UTC	
SzeTC465	1.66	1.33	0.73	0.41	0.16	0.86
Sze521	2.83	1.20	1.65	0.62	0.58	1.37
NS3014	3.44	2.02	1.92	0.61	0.30	1.66
Sarolta	3.28	2.83	2.70	0.87	0.16	1.97
NS6030	3.18	4.97	1.64	0.55	0.36	2.14
NS6102	4.82	3.42	2.12	0.30	0.13	2.16
Sze363	5.42	2.10	2.60	0.97	0.53	2.32
Kenéz	3.79	3.30	3.59	0.85	0.30	2.37
GKT288	4.07	2.17	4.84	1.09	0.15	2.46
NS5051	6.04	2.41	2.59	1.20	0.70	2.59
NS4015	6.87	2.79	2.87	0.81	0.76	2.82
NS640	8.32	3.71	1.89	0.47	0.21	2.92
Sze386	5.38	3.08	4.08	1.37	1.00	2.98
NS6010	11.73	5.42	4.33	0.32	0.16	4.39
Sze349	9.85	5.16	6.60	0.76	0.31	4.53
NS4023	12.27	2.90	7.01	0.38	0.25	4.56
SzeTC367	8.41	5.71	7.95	1.52	0.79	4.88
GK Boglár	13.59	4.56	6.43	0.90	0.49	5.19
NS5043	17.92	6.31	4.97	0.99	0.89	6.22
NS4030	15.96	8.00	7.33	0.57	0.21	6.41
Mean	7.44	3.67	3.89	0.78	0.42	3.24
LSD 5%	2.96	1.14	1.59	0.52	0.47	0.76
**Correlations**	**Fg**	**Fc**	**Fv**	**Afl**	**K**	
Fc	0.7686 ***					
Fv	0.7182 ***	0.6502 **				
Afl	−0.0129	0.0282	0.3194			
Check	0.1712	0.0589	0.1387	0.7181 ***		
Mean	0.9584 ***	0.8540 ***	0.8573 ***	0.1541	0.2169	

Fg ^x^ = *F. graminearum*, Fc = *F. culmorum*, Fv = *F. verticillioides*, Af = *A. flavus*. *** *p* = 0.001, ** *p* = 0.01. Highlighted: data: lower than mean for the given trait, UTC = untreated control, natural infection.

**Table 2:** Five-way ANOVA of the ear coverage data of the maize resistance tests against toxigenic fungi in 2012–2013.

A/ANOVA					
Source of Variance	SS	df	MS	F	
Hybrid A	7810.85	19	411.10	34.11 ***	
Isolate B	178.40	1	178.40	14.80 ***	
Year C	4855.54	1	4855.54	402.94 ***	
Location D	4303.72	1	4303.72	357.15 **	
Toxic spp. E	20,654.59	4	5163.65	428.51 ***	
A × B	1570.65	19	82.67	6.86 **	
A × C	3738.80	19	196.78	16.33 ***	
A × D	5517.86	19	290.41	24.10 ***	
A × E	10,541.69	76	138.71	11.51 ***	
B × C	21,136.66	1	21,136.66	1754.07 ***	
B × D	76.63	1	76.63	6.35 *	
B × E	10,117.77	4	2529.44	209.91 ***	
C × D	301.22	1	301.22	24.99 ***	
C × E	11,110.72	4	2777.68	230.51 ***	
D × E	3495.71	4	873.93	72.52 ***	
Interactions 3–5	55,174	625	88.28	7.32 **	
Within	28,928.70	2400	12.05		
Total	189,513.61	3199			
**Comparison of the main effects with two-ways interactions, based on MS values from Table 3**					
A × B df 19, 19	Hybrid A/AB			4.97	***
A × B df 1, 19	Isolate B/AB			2.16	ns
A × C df 19,19	Hybrid A/AC			2.09	ns
A × C df 1, 19	Year C/AC			24.67	***
AD df 19,	Hybrid A/AD			1.42	ns
AD df 1, 19	Location D/AD			14.82	**
AE df 19,	Hybrid A/AD			2.96	*
AE df 1, 19	Toxic spp. E/AD			37.23	***

*** *p* = 0.001, ** *p* = 0.01, * *p* = 0.05, ns = non significant.

**Table 3:** Maize ear rot resistance tests, separate ANOVAs for the different toxic species, 2012–2013.

**Source**	**MQ Values**					
**Variance**	**df**	**Fg ^x^**	**Fc**		**Fv**	**Af**
Hybrid A	19	703.48 ***	102.57 ***		153.58 ***	3.82 **
Location B	1	4321.77 ***	1108.22 ***		2367.66 ***	0.11 ns
Year C	1	9983.15 ***	831.57 ***		5058.64 ***	40.44 ***
Isolate D	1	3794.60 ***	1466.87 ***		5033.99 ***	0.84 ns
**A × B**	**19**	490.87 ***	127.32 ***		131.75 ***	1.98 **
**A × C**	**19**	435.06 ***	134.70 ***		123.62 ***	2.10 **
**A × D**	**19**	196.02 ***	110.56 ***		145.67 ***	**1.27 ns**
B × C	1	168.97 **	755.86 ***		2765.34 ***	1.87 ns
B × D	1	1307.89 ***	1643.06 ***		3503.68 ***	4.2 *
C × D	1	268.12 ***	301.84 ***		4354.64 ***	0.12 ns
A × B × C	19	308.59 ***	209.82 ***		120.96 ***	2.26 **
A × B × D	19	156.68 **	98.61 ***		127.69 ***	1.48 ns
A × C × D	19	66.88	162.30 ***		123.83 ***	1.25 ns
B × C × D	1	75.14	1276.80 ***		3344.70 ***	0.14 ns
A × B × C × D	19	49.24 *	108.99 ***		114.86 ***	2.31 **
Within	480	36.7	10.90		10.61	1.12
Total	639					
**Comparison of the main effects to two-way interactions, based on MS values**						
**Interaction**	**Compared**		**Fg**	**Fc**	**Fv**	**Af**
A × B df 19, 19	Hybrid A/AB		1.43	0.81	1.17	1.93
A × B df 1, 19	Isolate B/AB		8.80 **	8.70 **	17.99 ***	0.06
A × C df 19,19	Hybrid A/AC		1.62	0.76	1.24	1.82
A × C df 1, 19	Year C/AC		22.94 ***	6.17 *	40.92 ***	0.21
AD df 19, 19	Hybrid A/AD		3.58 ***	0.93	1.05	3 **
AD df 1, 19	Location D/AD		19.35 ***	13.26 **	34.55 ***	0.66

*** *p* = 0.001, ** *p* = 0.01, * *p* = 0.05, ns = non significant. ^x^ Fg = *F. graminearum*, Fc = *F. culmorum*, Fv = *F. verticillioides*, Af = *A. flavus*, **Bold**: two-way interactions containing the hybrid component.

**Table 4:** Correlations between resistance reaction of maize hybrids against toxigenic species and their isolates across years and locations, 2012–2013.

Correlations	Fg 13.38	Fg 13.05	Fc 12,551.2	Fc 12,375.2	Fv 18TE	Fv SZ 1.1.1	Af 126	Af 171
Fg ^x^ 13.05	0.722 ***							
Fc 12,551.2	0.392	0.156						
Fc 12,375.2	0.463 *	0.769 ***	**−0.079**					
Fv 18TE	0.7674 ***	0.674 **	0.546 *	0.533 *				
Fv SZ 1.1.1	0.108	−0.270	0.341	−0.092	**0.111**			
Afl Af 126	0.047	−0.385	0.515	−0.299	0.090	0.729 ***		
Afl Af 171	0.410	0.033	0.162	0.121	0.296	0.546 *	0.530 *	
Check	0.316	0.085	0.078	0.040	0.081	0.498	0.405	0.790 ***

*** *p* = 0.001, ** *p* = 0.01, * *p* = 0.05. **Bold**: Correlations between isolates of the same toxigenic species, ^x^ Fg = *F. graminearum*, Fc = *F. culmorum*, Fv = *F. verticillioides*, Af = *A. flavus.*

**Table 5:** Resistance of maize to toxigenic fungi, ranks (1–20) and their variance for ear coverage across isolates, years and locations, 2012–2013.

Hybrids			Toxic Species, Mean of Isolates				Variance
	Fg	Fc	Fv	Af	UTC	Mean	
SzeTC465	1.0	2.0	1.0	4.0	3.0	2.2	1.7
NS6102	8.0	12.0	6.0	1.0	1.0	5.6	22.3
NS3014	5.0	3.0	5.0	8.0	9.0	6.0	6.0
Sze521	2.0	1.0	3.0	9.0	15.0	6.0	35.0
Sarolta	4.0	8.0	9.0	13.0	4.0	7.6	14.3
NS6030	3.0	15.0	2.0	6.0	12.0	7.6	32.3
NS640	13.0	13.0	4.0	5.0	7.0	8.4	18.8
GKT288	7.0	5.0	14.0	17.0	2.0	9.0	39.5
Kenéz	6.0	11.0	11.0	12.0	10.0	10.0	5.5
Sze363	10.0	4.0	8.0	15.0	14.0	10.2	20.2
NS6010	16.0	17.0	13.0	2.0	5.0	10.6	45.3
NS4023	17.0	9.0	18.0	3.0	8.0	11.0	40.5
NS4015	12.0	7.0	10.0	11.0	17.0	11.4	13.3
NS5051	11.0	6.0	7.0	18.0	16.0	11.6	28.3
Sze349	15.0	16.0	17.0	10.0	11.0	13.8	9.7
Sze386	9.0	10.0	12.0	19.0	20.0	14.0	26.5
NS4030	19.0	20.0	19.0	7.0	6.0	14.2	49.7
GK Boglár	18.0	14.0	16.0	14.0	13.0	15.0	4.0
NS5043	20.0	19.0	15.0	16.0	19.0	17.8	4.7
SzeTC367	14.0	18.0	20.0	20.0	18.0	18.0	6.0
Mean	10.5	10.5	10.5	10.5	10.5	10.5	21.2
**Correlations**	**Fg**	**Fc**	**Fv**	**Af**	**Check**	**Mean**	
Fc	0.7007 ***						
Fv	0.7759 ***	0.6015 **					
Af	0.0902	−0.0301	0.3338				
Check	0.2391	0.1038	0.1759	0.6390 **			
Mean	0.8013 ***	0.6785 ***	0.8245 ***	0.5806 **	0.6162 **		

*** *p* = 0.001, ** *p* = 0.01. ^x^ Fg = *F. graminearum*, Fc = *F. culmorum*, Fv = *F. verticillioides*, Afl = *A. flavus*, highlighted: ranks between 1 and 10. UTC: untreated control, natural infection.

**Table 6:** Toxin contamination following artificial inoculation of ear rot pathogens in maize hybrids, across years, locations and isolates, 2012–2013. Unit: mg/kg. LSD data were from the independent toxin analyses and those of the general mean.

Hybrid	Toxigenic spp.				Mean
	Fg	Fc	Fc/Fg	Fv	
SzeTC465	2.45	2.31	2.38	4.41	3.06
NS3014	3.56	1.85	2.70	4.56	3.32
NS6102	3.97	3.15	3.56	2.88	3.34
NS4015	6.14	2.53	4.34	3.84	4.17
Sze386	6.19	2.21	4.20	5.50	4.63
Sarolta	3.07	3.18	3.12	8.71	4.98
NS6030	2.34	4.52	3.43	10.91	5.92
NS640	6.55	5.60	6.08	5.90	6.02
Sze349	8.86	4.55	6.70	7.07	6.83
GKT288	4.47	3.22	3.85	15.95	7.88
GK Boglár	10.89	5.02	7.96	7.85	7.92
Sze363	2.76	1.83	2.29	19.21	7.93
NS4023	12.00	4.80	8.40	7.23	8.01
Sze521	2.75	1.19	1.97	20.47	8.14
NS4030	14.52	9.47	12	3.23	9.07
NS6010	12.05	6.07	9.06	10.77	9.63
NS5043	15.20	13.28	14.24	3.18	10.55
Kenéz	1.19	0.72	0.95	30.60	10.83
SzeTC367	8.46	5.76	7.11	28.67	14.29
NS5051	3.49	3.06	3.28	43.83	16.79
Mean	6.55	4.22	5.38	12.24	7.67
LSD 5%	2.10	2.96	1.82	7.74	0.48
**Correlations**	**Fg**	**Fc**	**Fg/Fc**	**Fv**	
Fc	0.8463 ***				
Fc/Fg	0.9745 ***	0.9441 ***			
Fv	−0.3728	−0.3130	−0.3623		
Mean	0.2547	0.2982	0.2830	0.7903 **	

*** *p* = 0.001, ** *p* = 0.01. ^x^ Fg = *F. graminearum*, Fc = *F. culmorum*, Fv = *F. verticillioides*, Afl = *A. flavus*, highlighted: data lower than average.

**Table 7:** Resistance of maize to toxigenic fungi; ANOVA for the toxin analyses individually, Szeged-Kupusina 2012–2013.

	df	DON Fg ^x^		DON Fc		Fum B1 + B2 Fv	
		MQ	F	MQ	F_A × B × C × D_	MQ	F
Hybrid A	19	606.1	16.49 ***	286.58	3.49 ***	3957.44	15.78 ***
Location B	1	23,797.4	647.72 ***	5671.60	69.25 ***	79,913.85	318.72 ***
Year C	1	8571.7	233.30 ***	5.29	0.06	3726.13	14.86 ***
Isolate D	1	1160.6	31.58 ***	2193.39	26.78 ***	165.78	0.66
A × B	19	602.8	16.40 ***	193.32	2.36 **	3956.58	15.78 ***
A × C	19	358.2	9.74 ***	184.02	2.24 **	1661.05	6.62 ***
A × D	19	154.5	4.20 ***	203.14	2.48 **	1431.70	5.71 ***
B × C	1	7425.0	202.09 ***	18.19	0.22	3308.08	13.19 ***
B × D	1	578.2	15.73 ***	3675.49	44.88 ***	8.45	0.03
C × D	1	146.5	3.98 *	120.03	1.47	12.06	0.05
A × B × C	19	326.3	8.88 ***	176.99	2.16 **	1704.11	6.79 ***
A × B × D	19	145.5	3.95 ***	273.78	3.34 **	1433.99	5.71 ***
A × C × D	19	222.0	6.04 ***	83.07	1.01	2485.15	9.91 ***
B × C × D	1	393.2	10.70 ***	182.93	2.23	0.46	0.00
A × B × C × D	19	200.8	5.46 ***	81.89		2559.08	10.20 ***
Within	480	36.7		18.47		250.73	
Total	639						

*** *p* = 0.001, ** *p* = 0.01, * *p* = 0.05. ^x^ Fg = *F. graminearum*, Fc = *F. culmorum*, Fv = *F. verticillioides*, Afl = *A. flavus*.

**Table 8:** Toxin contamination following artificial inoculation of ear rot pathogens in maize hybrids across years, locations and isolates, ranks, 2012–2013.Unit: ranges between 1–20.

Hybrid	Toxigenic Spp. Ranks 1–20				Mean	Variance
	Fg	Fc	Fc/Fg	Fv		
Sze521	4	2	2	17	7.67	66.3
Kenéz	1	1	1	19	7.00	108.0
SzeTC465	3	6	4	5	4.67	**2.3**
NS6102	9	9	9	1	6.33	21.3
NS3014	8	4	5	6	6.00	**4.0**
NS6030	2	12	8	14	9.33	41.3
Sze363	5	3	3	16	8.00	49.0
NS4015	11	7	12	4	7.33	12.3
Sarolta	6	10	6	12	9.33	**9.3**
Sze386	12	5	11	7	8.00	13.0
NS640	13	16	13	8	12.33	16.3
GKT288	10	11	10	15	12.00	**7.0**
NS5051	7	8	7	20	11.67	52.3
Sze349	15	13	14	9	12.33	**9.3**
NS4023	17	14	17	10	13.67	12.3
NS4030	19	19	19	3	13.67	85.3
NS6010	18	18	18	13	16.33	8.3
GK Boglár	16	15	16	11	14.00	**7.0**
NS5043	20	20	20	2	14.00	108.0
SzeTC367	14	17	15	18	16.33	**4.3**
Mean	10.5	10.5	10.5	10.5	10.5	31.9
	**Fg**	**Fc**	**Fc/Fg**	**Fv**		
Fc	0.8135 ***					
Fc/Fg	0.9563 ***	0.9067 ***				
Fv	−0.4211	−0.2676	−0.4045			
Mean	0.7724 ***	0.8575 ***	0.8091 ***	0.1726		

*** *p* = 0.001. ^x^ Fg = *F. graminearum*, Fc = *F. culmorum*, Fv = *F. verticillioides*, Afl = *A. flavus.* Highlighted: data lower than average. **Bold:** Rather uniform performance against different toxigenic fungi.

**Table 9:** Ear rot resistance of maize to 2-2 isolates of four toxigenic species and correlations between toxin responses of the isolates of toxigenic species from Figure 3, 2012–2013.

Correlations	Fv 18TE ^a^	Fv Sz1.1.	Fg 13.38	Fg 13.05	Fc 12375	Fc 12551
Fv Sz1.1.	0.4862 *					
Fg 13.38	−0.2600	−0.4505 *				
Fg 13.05	−0.1440	−0.3806x	0.6065 **			
Fc 12375	−0.1986	−0.4384x	0.8549 ***	0.5869 **		
Fc 12551	0.1020	−0.1461	0.3250	0.5484 *	**0.2385**	
Mean	0.7855 ***	0.5205 *	0.1982	0.2963	0.2385	0.3553

*** *p* = 0.001, ** *p* = 0.01, * *p* = 0.05, x *p*= 0.1 ^a^ Fv = *F. verticillioides*, Fg = *F. graminearum*, Fc = *F. culmorum.* **Bold** numbers: They are the correlations between the two isolates belonging to the same fungal species.
